# Correction: Kaewchim et al. Neutralizing and Enhancing Epitopes of the SARS-CoV-2 Receptor-Binding Domain (RBD) Identified by Nanobodies. *Viruses* 2023, *15*, 1252

**DOI:** 10.3390/v15101979

**Published:** 2023-09-22

**Authors:** Kanasap Kaewchim, Kittirat Glab-ampai, Kodchakorn Mahasongkram, Thanatsaran Saenlom, Watayagorn Thepsawat, Monrat Chulanetra, Kiattawee Choowongkomon, Nitat Sookrung, Wanpen Chaicumpa

**Affiliations:** 1 Graduate Program in Immunology, Department of Immunology, Faculty of Medicine Siriraj Hospital, Bangkok 10700, Thailand; kanasap.kaw@alumni.mahidol.ac.th; 2 Center of Research Excellence in Therapeutic Proteins and Antibody Engineering, Department of Parasitology, Faculty of Medicine Siriraj Hospital, Bangkok 10700, Thailand; kittirat.gla@mahidol.edu (K.G.-a.); kodchakorn.mah@mahidol.ac.th (K.M.); thanatsaran.sae@mahidol.ac.th (T.S.); watayagorn.the@mahidol.edu (W.T.); monrat.chl@mahidol.ac.th (M.C.); nitat.soo@mahidol.ac.th (N.S.); 3 Department of Biochemistry, Faculty of Sciences, Kasetsart University, Bangkok 10900, Thailand; fsciktc@ku.ac.th; 4 Biomedical Research Incubator Unit, Department of Research, Faculty of Medicine Siriraj Hospital, Mahidol University, Bangkok 10700, Thailand

## Error in Figure

In the original publication [1], there was a mistake in Figure 6. The published Figure 6 is a duplicate of Figure 2B. The correct Figure 6 appears below. The authors state that the scientific conclusions are unaffected. This correction was approved by the Academic Editor. The original publication has also been updated. The images were correct in the proofreading version, but the images might have been mixed up when we checked the proof. We apologize for the error. 

## Figures and Tables

**Figure 6:**
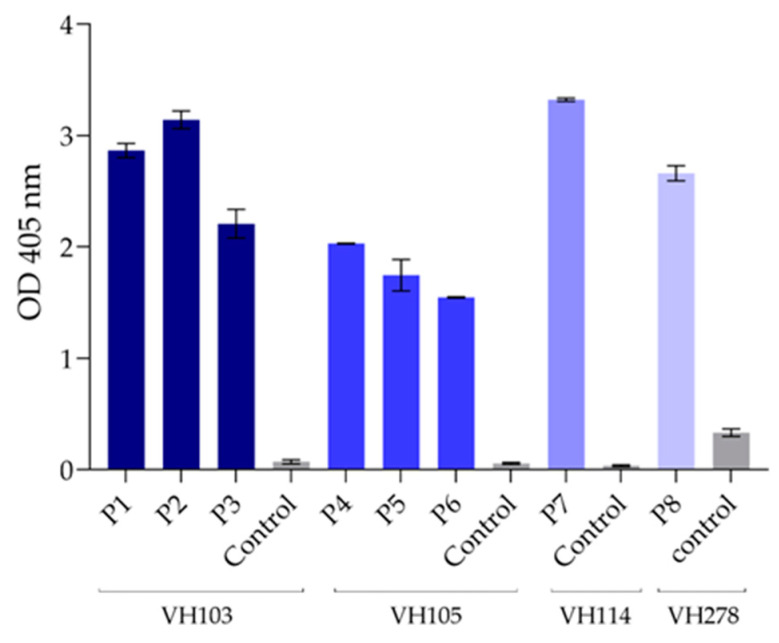
Peptide-binding ELISA to demonstrate binding of the nanobodies to the RBD peptides. The nanobodies VH103 bound to VH103-P1 (P1), VH103-P2 (P2), and VH103-P3 (P3); VH105 bound to VH105-P4 (P4), VH105-P5 (P5), and VH105-P6 (P6); VH114 bound to VH114-P7 (P7); and VH278 bound to VH278-P8 (P8), which verified that the consensus peptides contained epitopes of the respective nanobodies. Control (irrelevant) peptide was included in the assay as negative control.
