# Supplementary figures and images for: Neutralizing and Enhancing Epitopes of the SARS-CoV-2 Receptor-Binding Domain (RBD) Identified by Nanobodies
Source: Viruses. 2023 May 26;15(6):1252. doi: 10.3390/v15061252 (PMC10301551; doi:10.3390/v15061252)

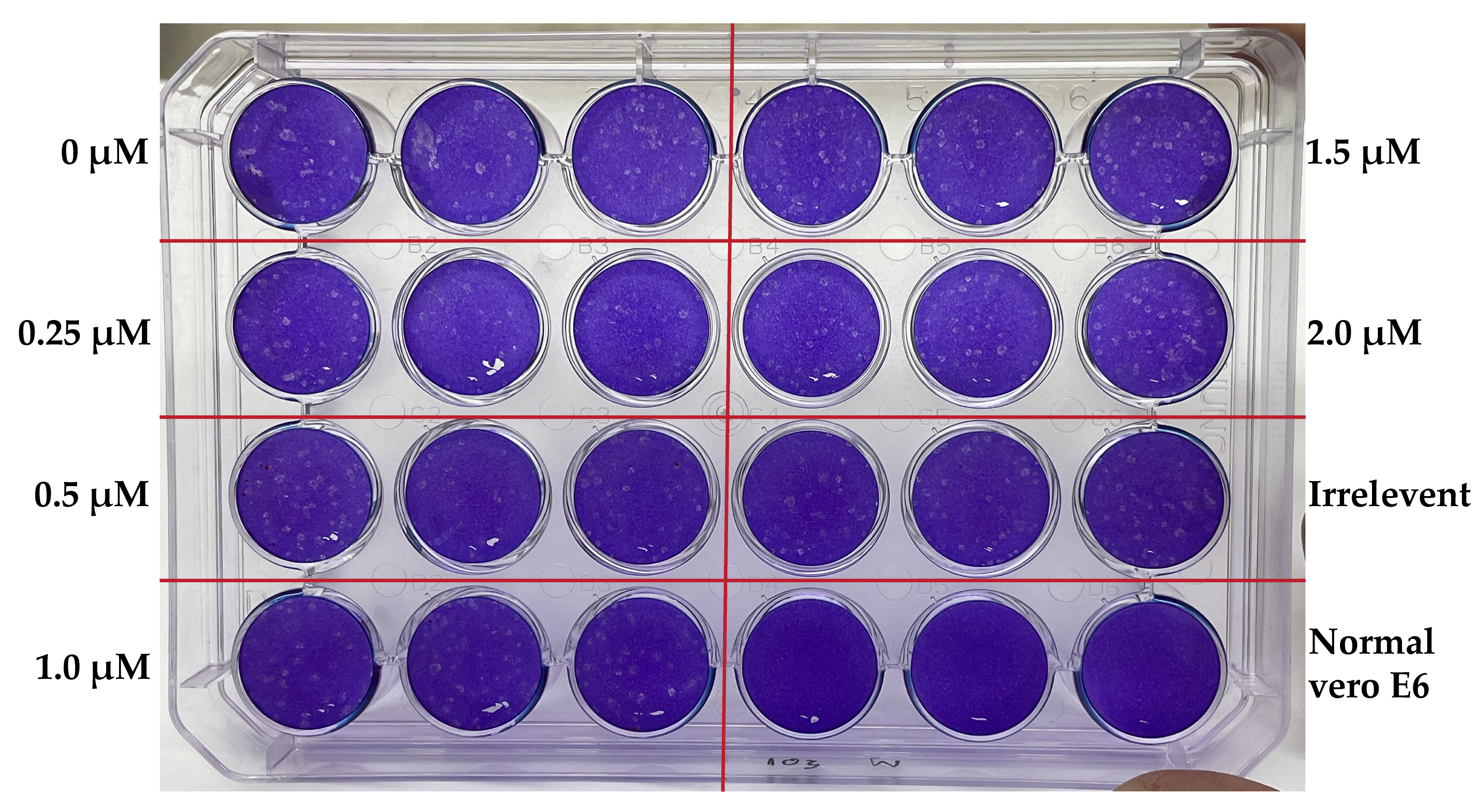

Supplement: Supplementary file 1 [file viruses-15-01252-s001.zip › 1. VH103 wuhan.tif]

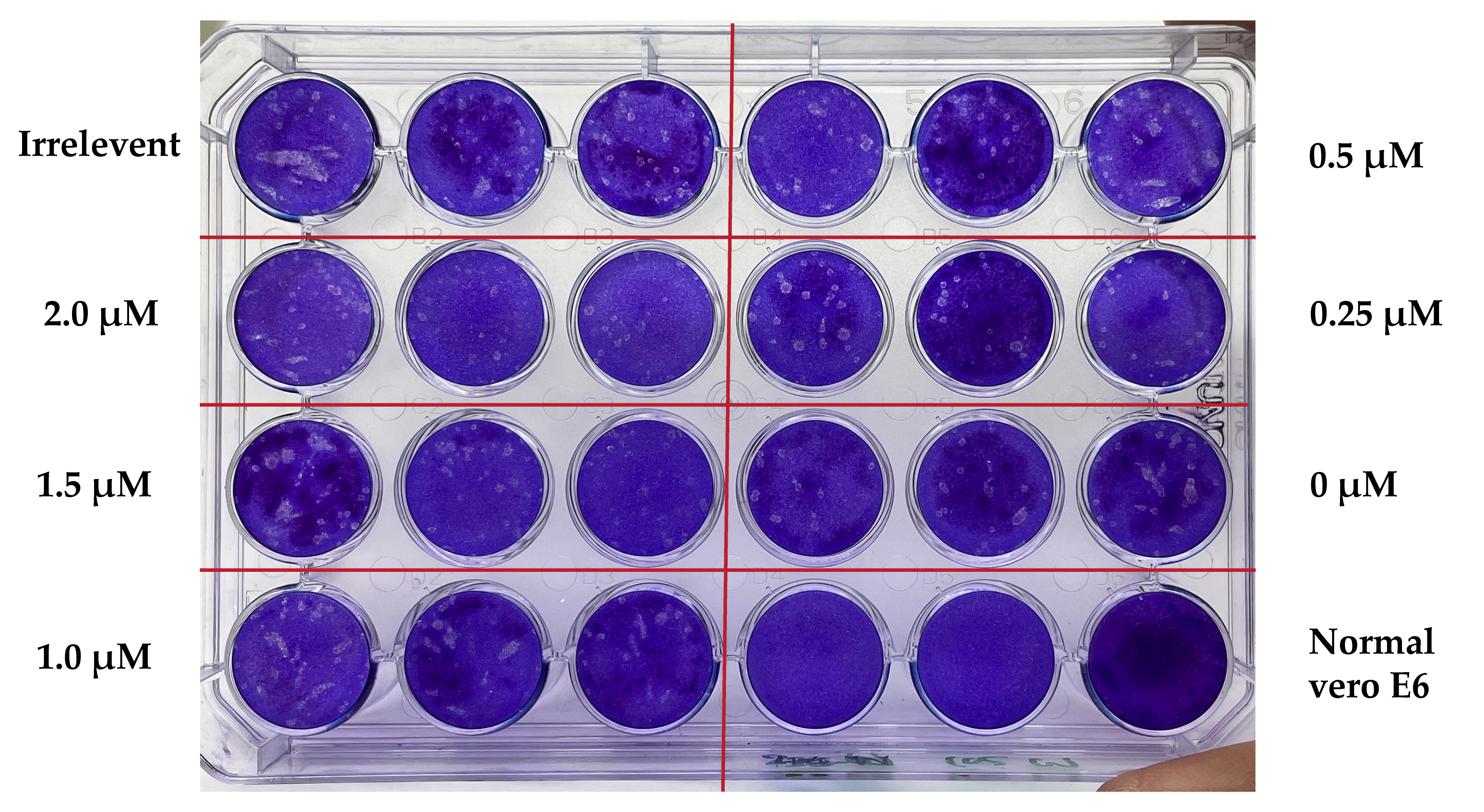

Supplement: Supplementary file 1 [file viruses-15-01252-s001.zip › 10. VH105 omicron.tif]

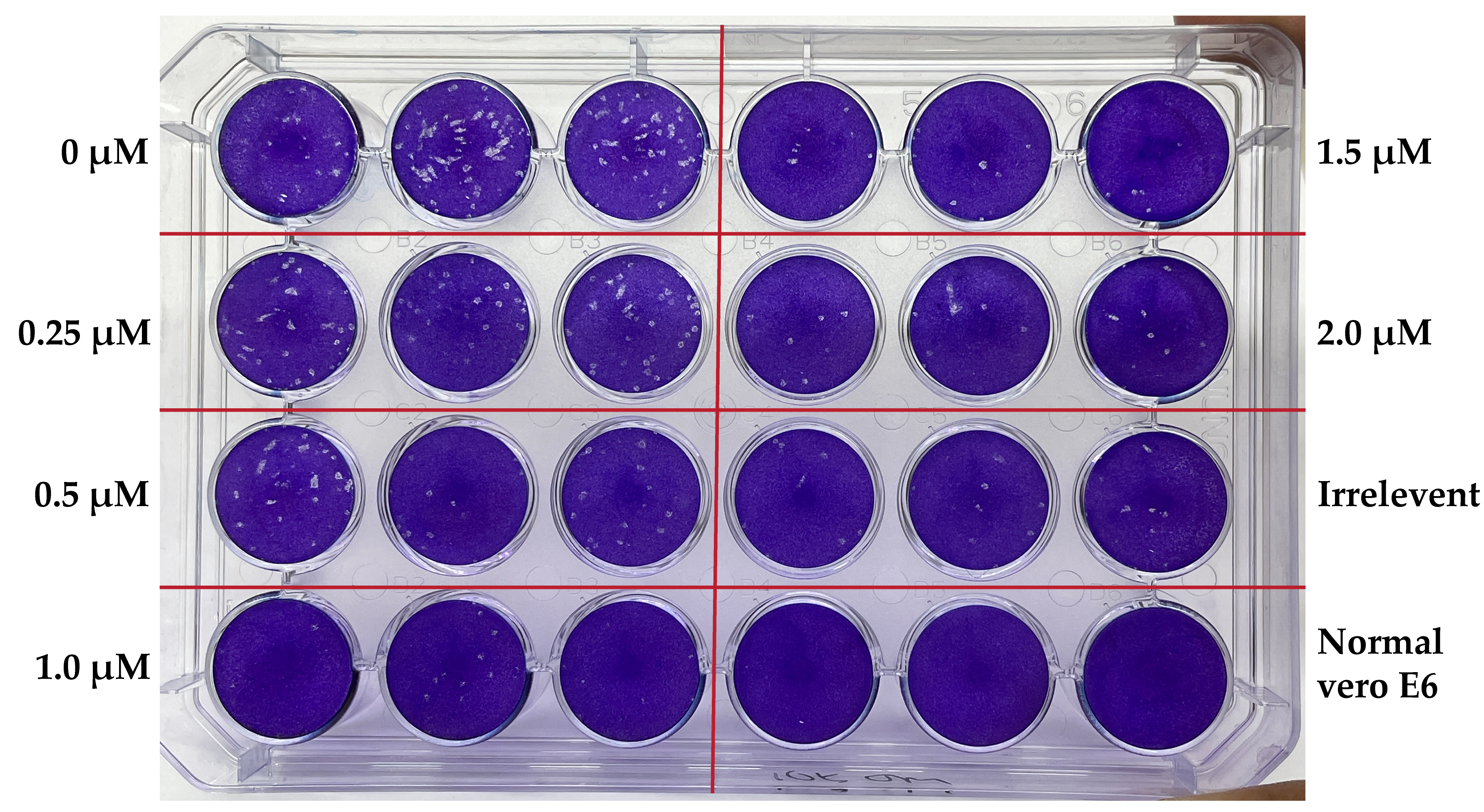

Supplement: Supplementary file 1 [file viruses-15-01252-s001.zip › 11. VH114 omicron.tif]

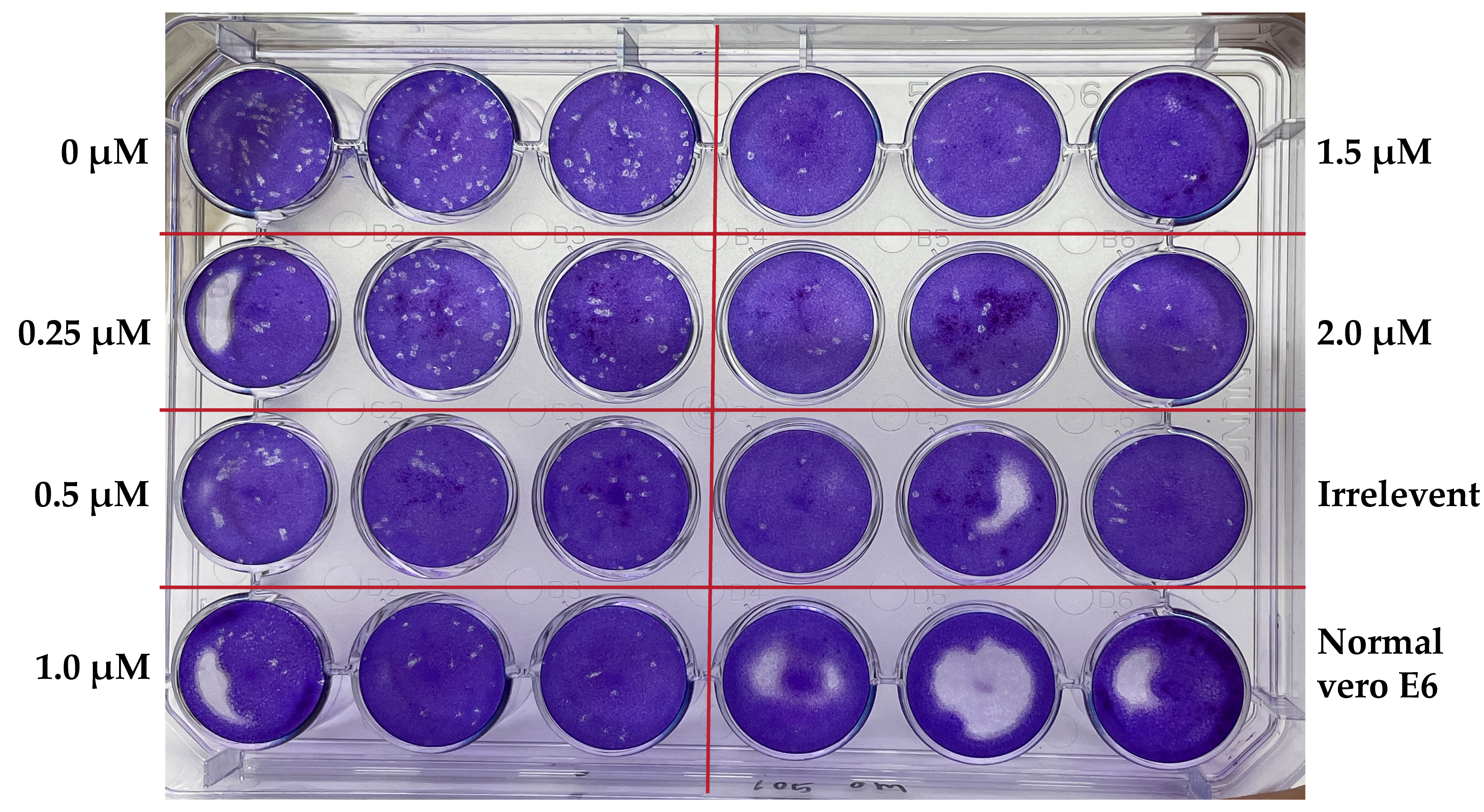

Supplement: Supplementary file 1 [file viruses-15-01252-s001.zip › 12. VH278 omicron.tif]

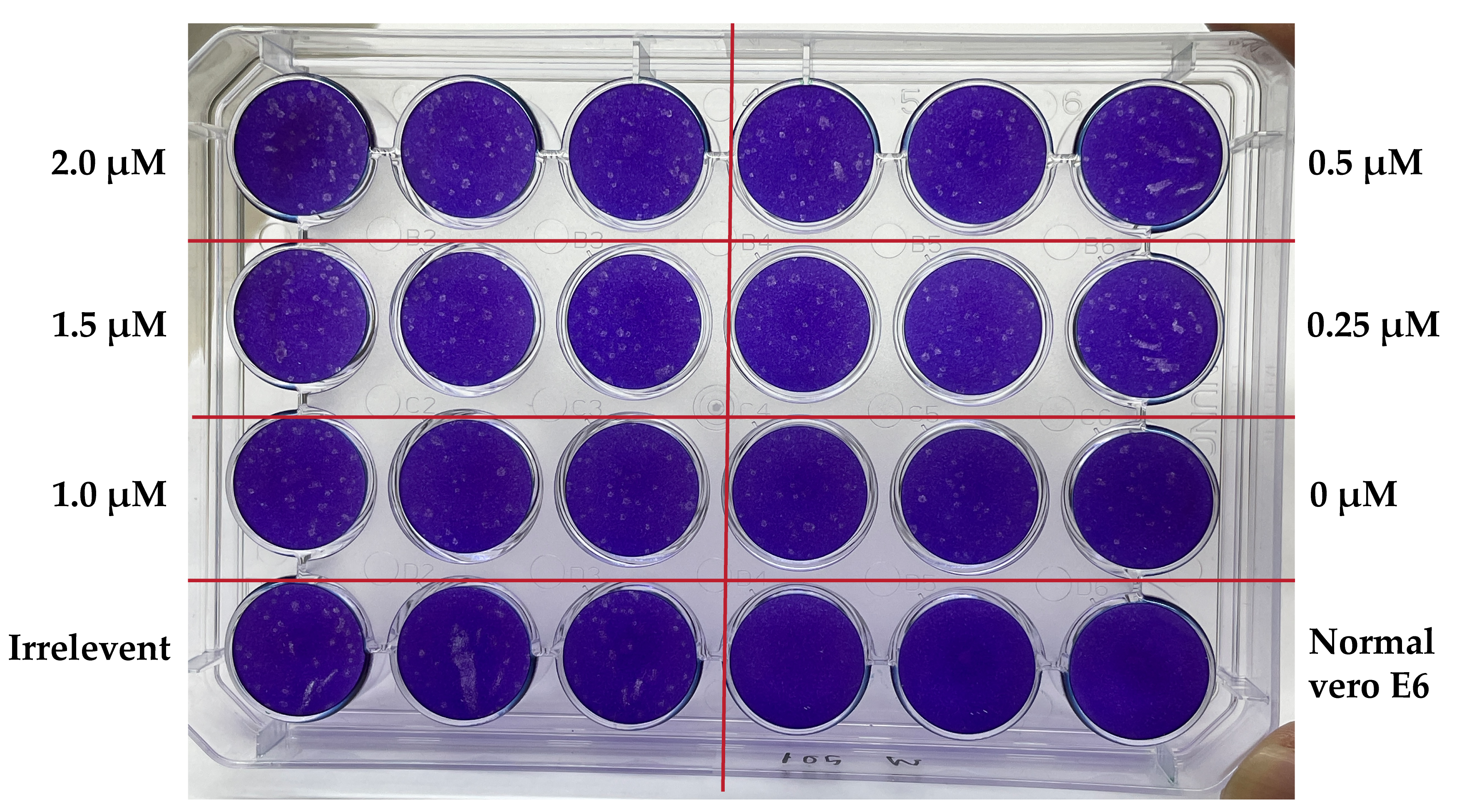

Supplement: Supplementary file 1 [file viruses-15-01252-s001.zip › 2. VH105 wuhan.tif]

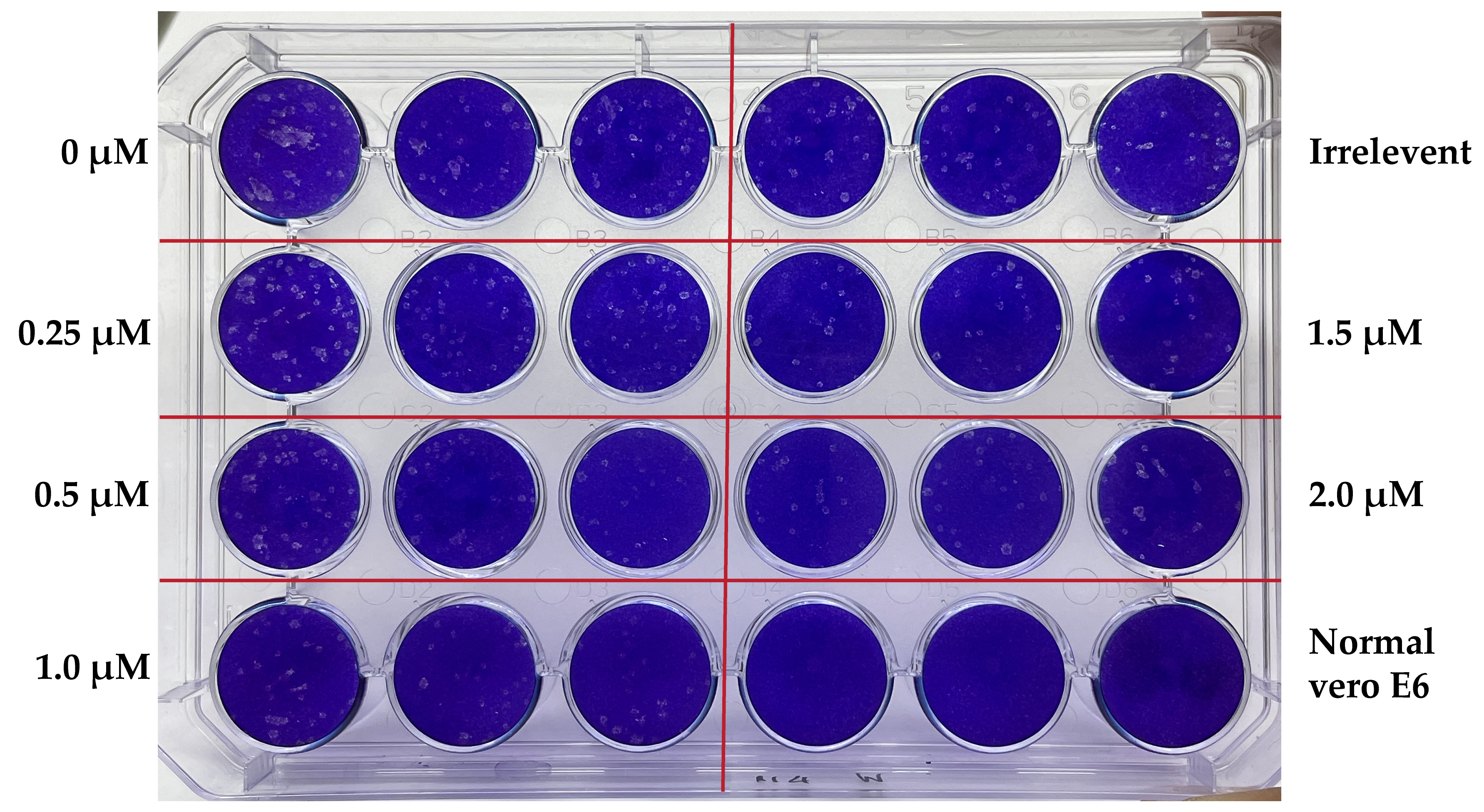

Supplement: Supplementary file 1 [file viruses-15-01252-s001.zip › 3. VH114 wuhan.tif]

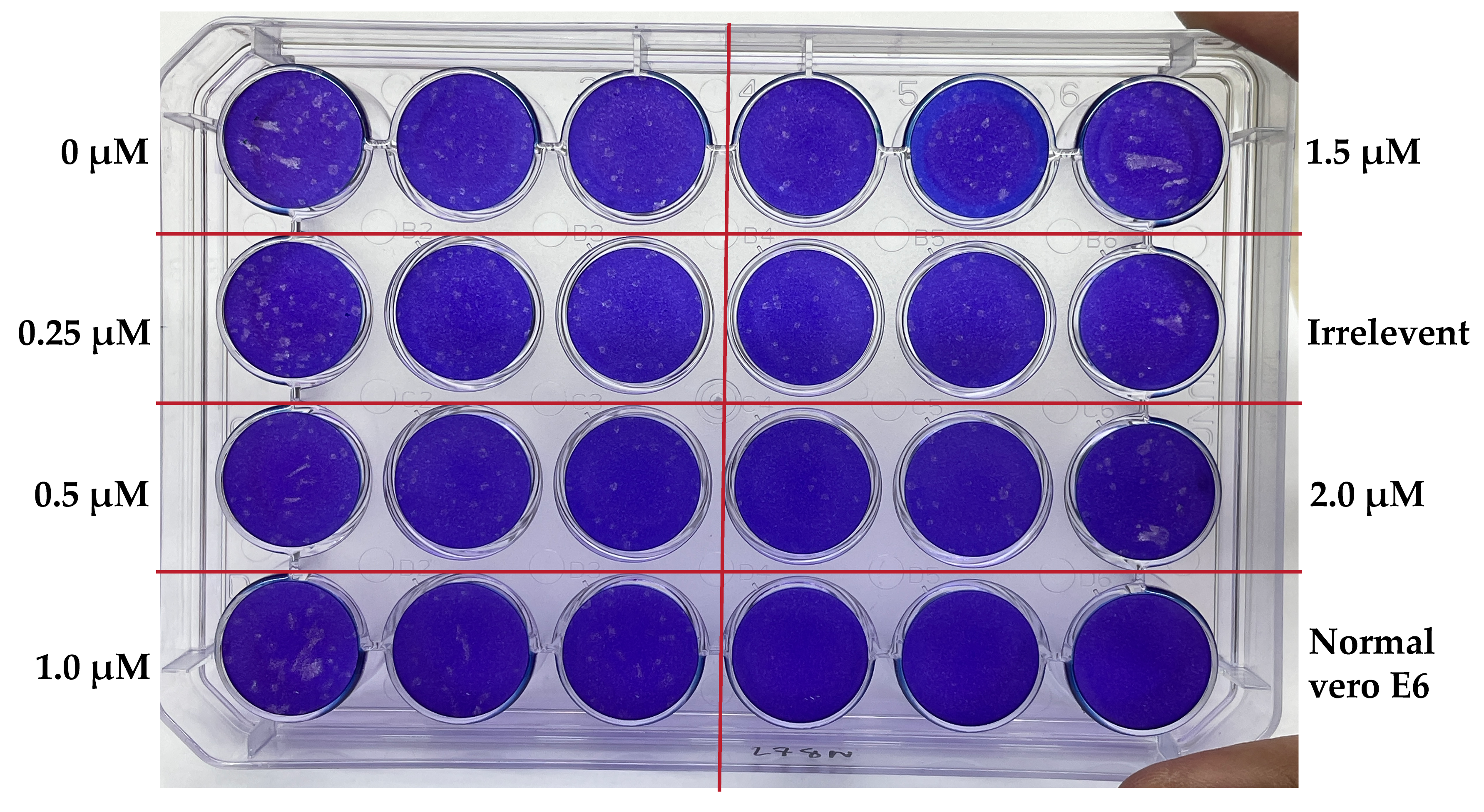

Supplement: Supplementary file 1 [file viruses-15-01252-s001.zip › 4. VH278 wuhan.tif]

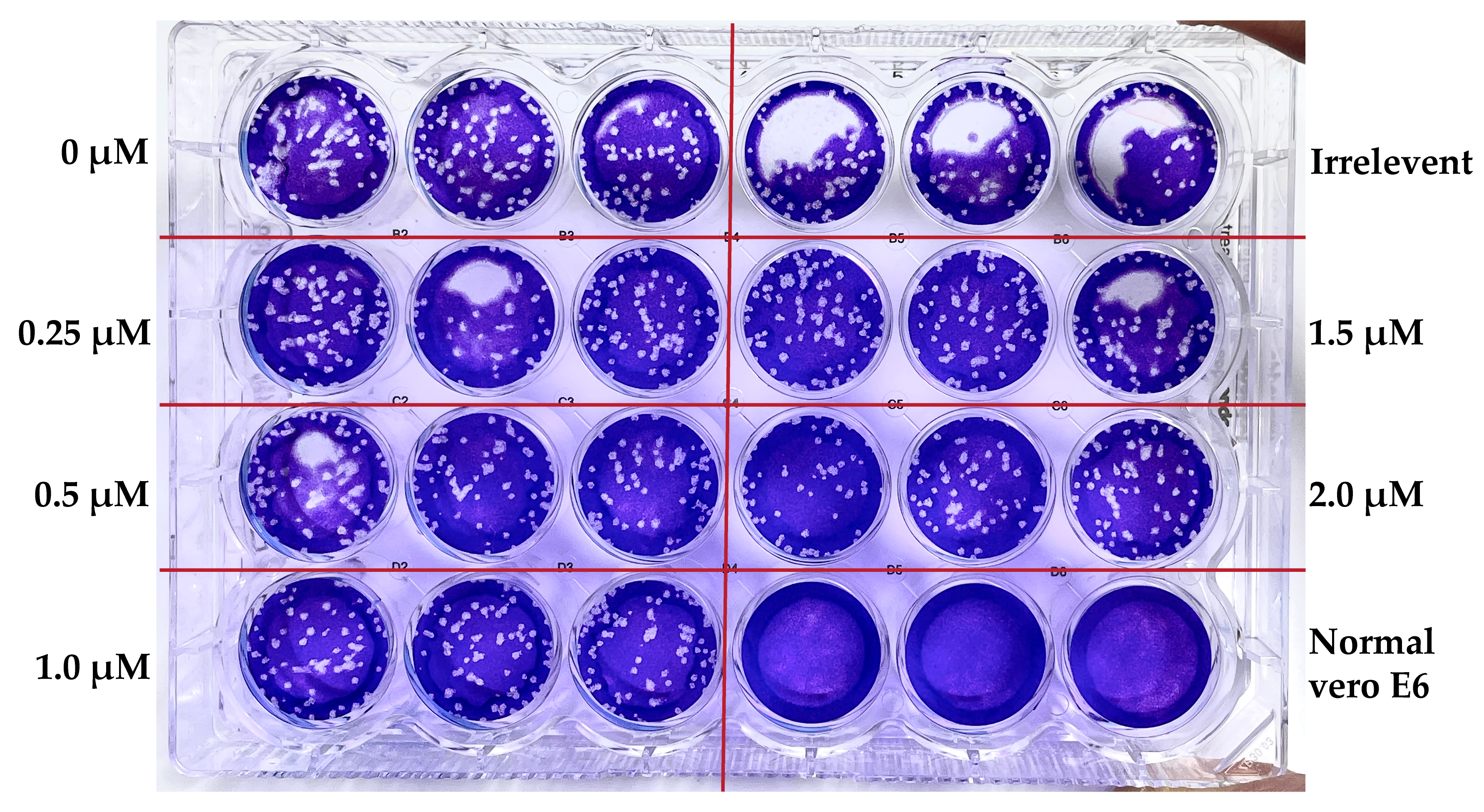

Supplement: Supplementary file 1 [file viruses-15-01252-s001.zip › 5. VH103 delta.tif]

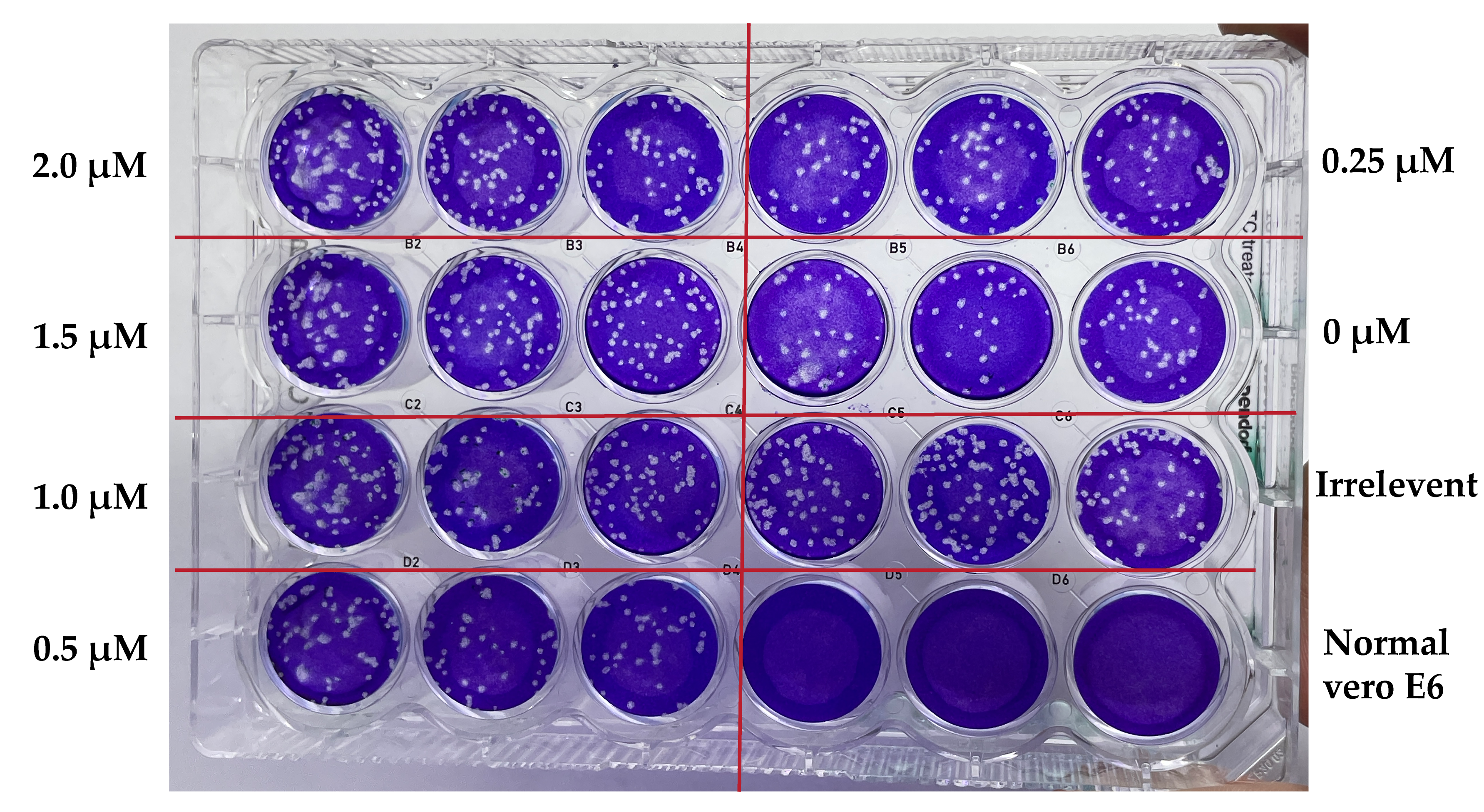

Supplement: Supplementary file 1 [file viruses-15-01252-s001.zip › 6. VH105 delta.tif]

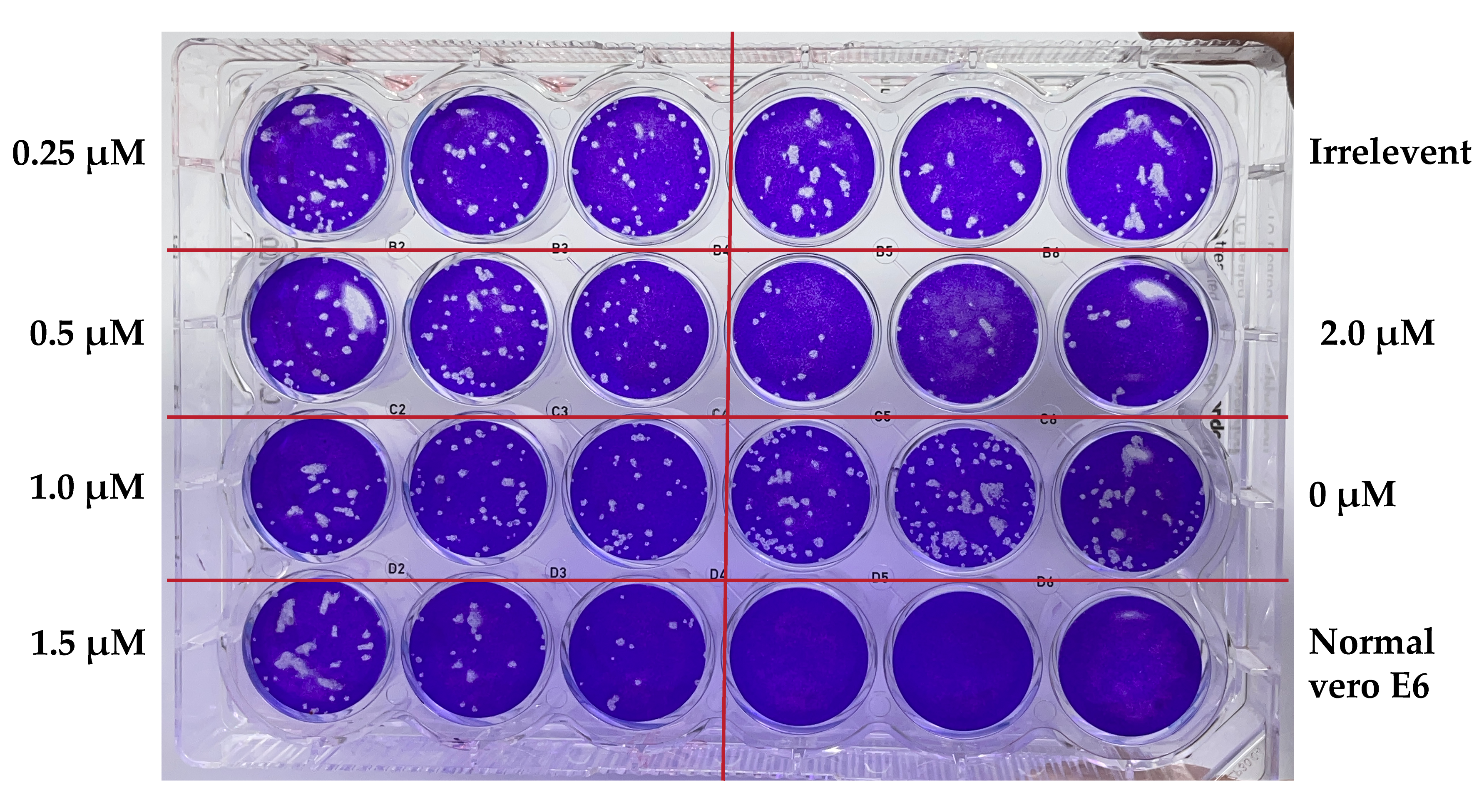

Supplement: Supplementary file 1 [file viruses-15-01252-s001.zip › 7. VH114 delta.tif]

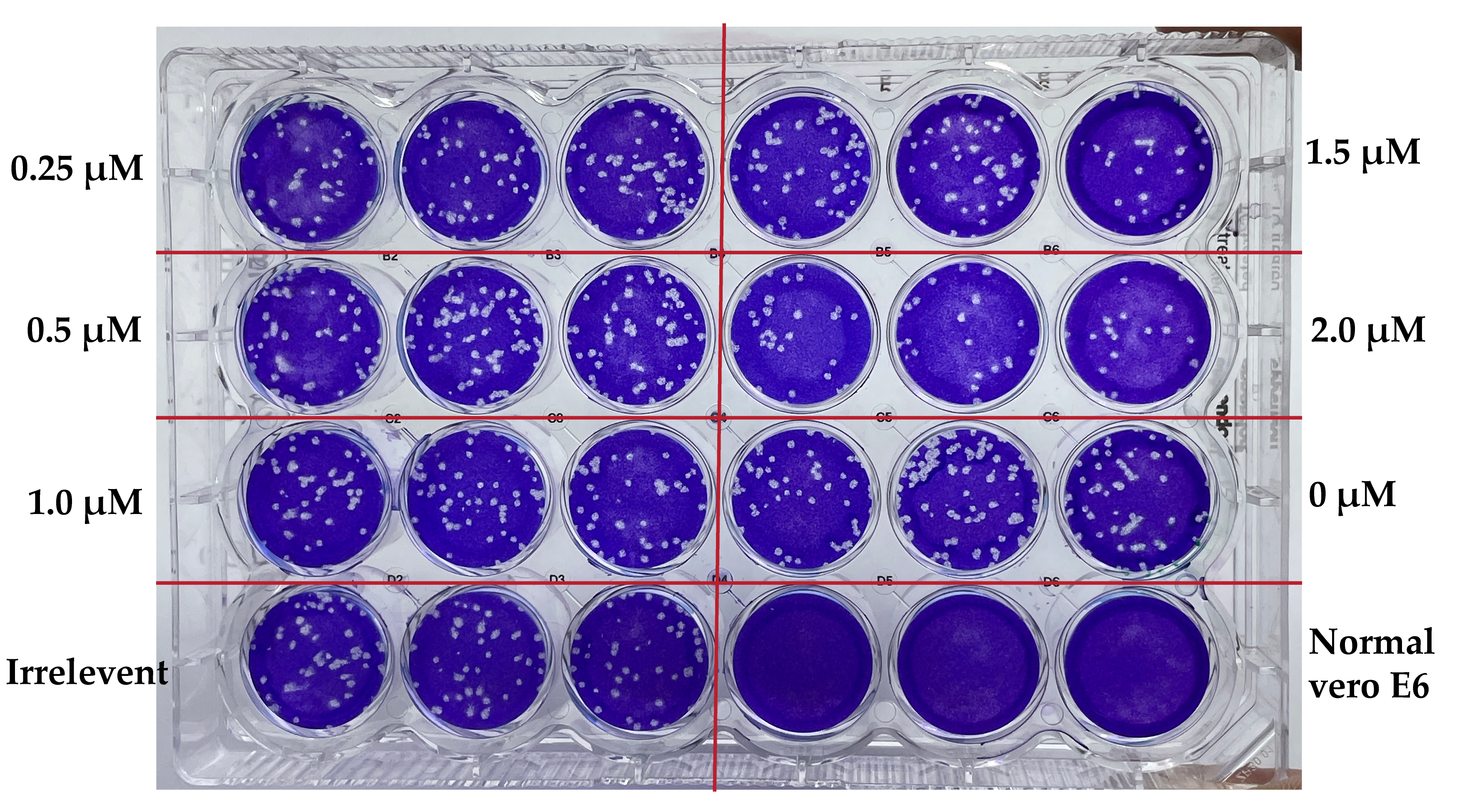

Supplement: Supplementary file 1 [file viruses-15-01252-s001.zip › 8. VH278 delta.tif]

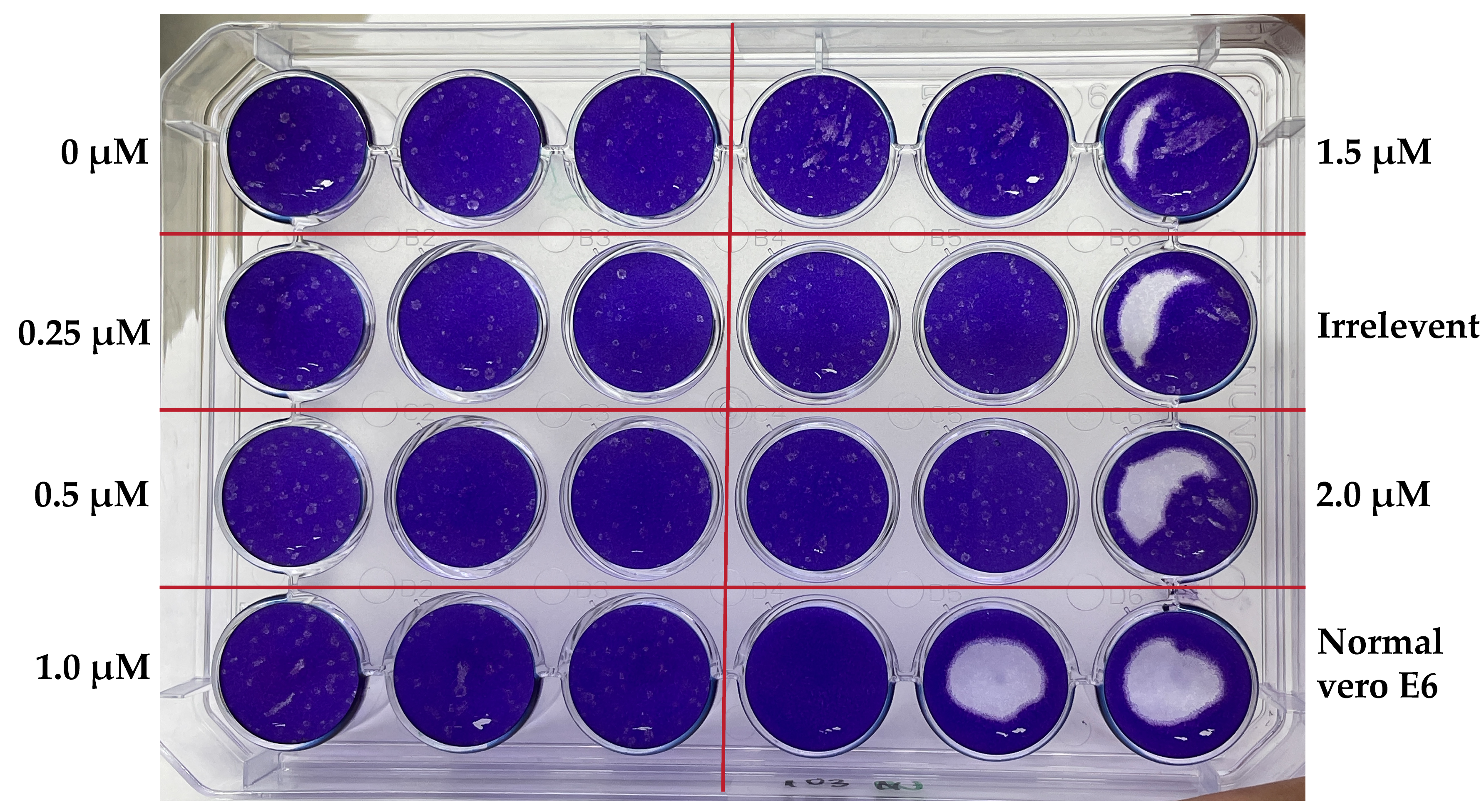

Supplement: Supplementary file 1 [file viruses-15-01252-s001.zip › 9. VH103 omicron.tif]

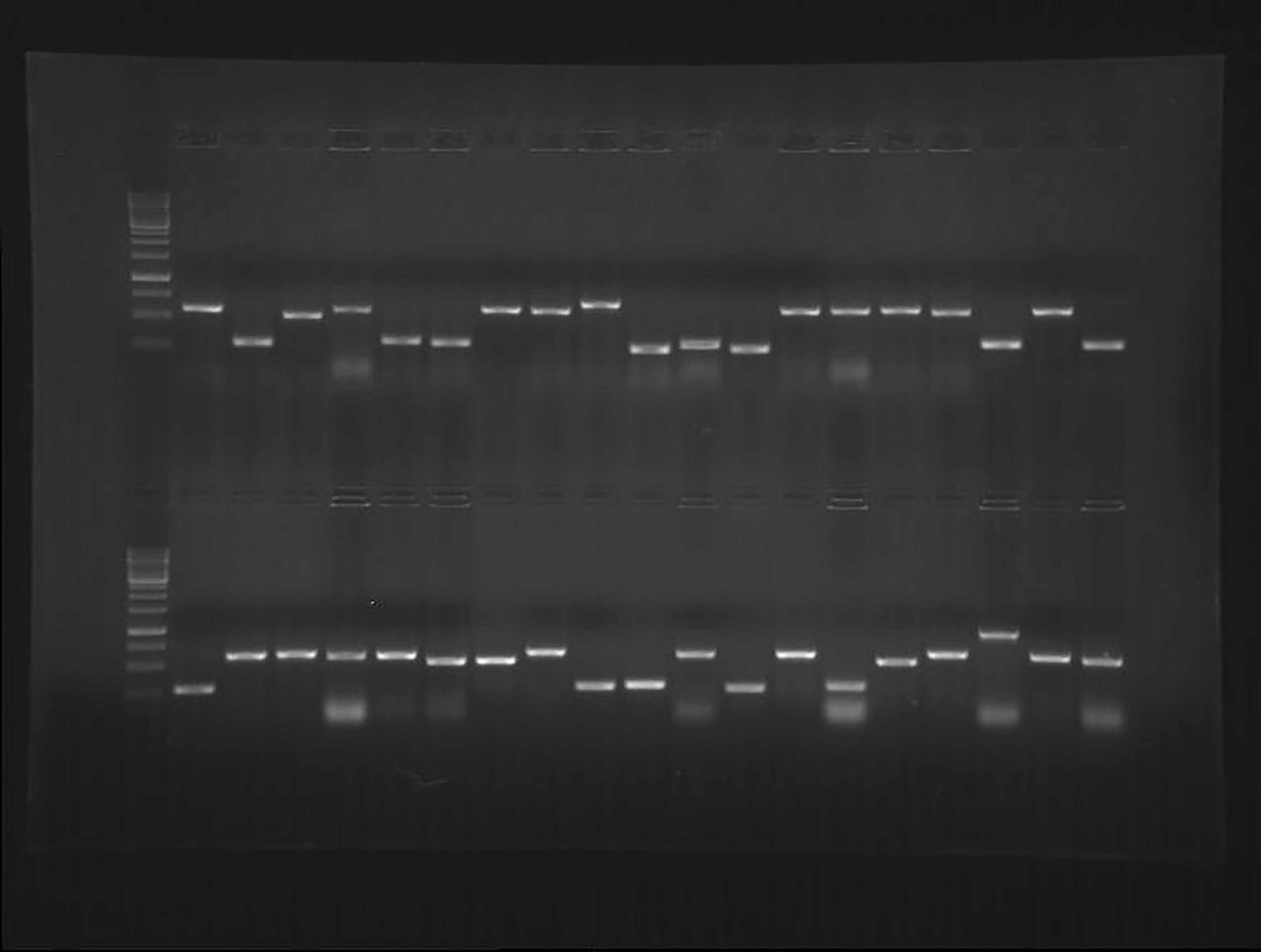

Supplement: Supplementary file 1 [file viruses-15-01252-s001.zip › Unprocessed 1A.tif]

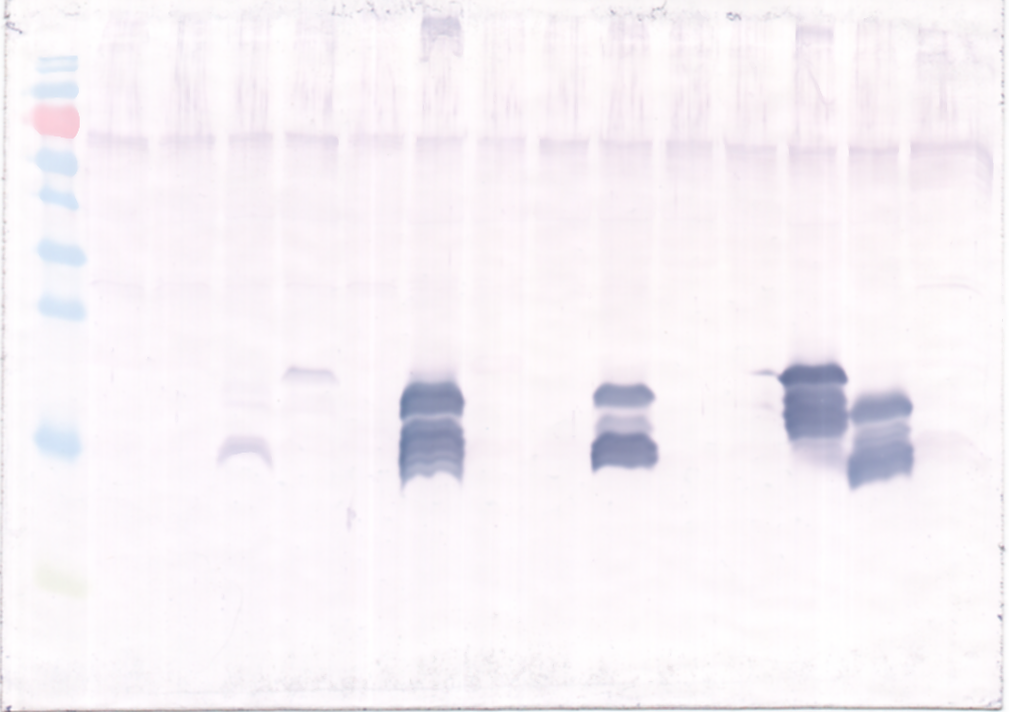

Supplement: Supplementary file 1 [file viruses-15-01252-s001.zip › Unprocessed 1B.tiff]

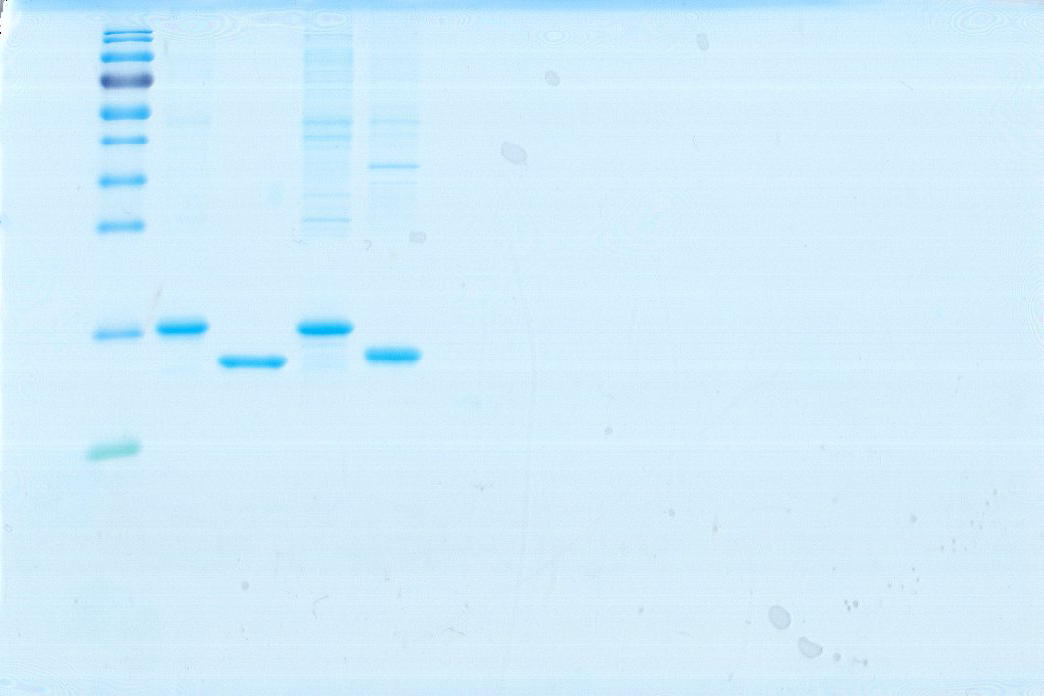

Supplement: Supplementary file 1 [file viruses-15-01252-s001.zip › Unprocessed 2A.tif]
